# Components of smartphone cognitive-behavioural therapy for subthreshold depression among 1093 university students: a factorial trial

**DOI:** 10.1136/ebmental-2022-300455

**Published:** 2022-05-16

**Authors:** Masatsugu Sakata, Rie Toyomoto, Kazufumi Yoshida, Yan Luo, Yukako Nakagami, Teruhisa Uwatoko, Tomonari Shimamoto, Aran Tajika, Hidemichi Suga, Hiroshi Ito, Michihisa Sumi, Takashi Muto, Masataka Ito, Hiroshi Ichikawa, Masaya Ikegawa, Nao Shiraishi, Takafumi Watanabe, Ethan Sahker, Yusuke Ogawa, Steven D Hollon, Linda M Collins, Edward R Watkins, James Wason, Hisashi Noma, Masaru Horikoshi, Taku Iwami, Toshi A Furukawa

**Affiliations:** 1 Department of Health Promotion and Human Behavior, Kyoto University Graduate School of Medicine/School of Public Health, Kyoto, Japan; 2 Kyoto University Health Service, Kyoto, Japan; 3 Department of Psychiatry, Kyoto University Hospital, Kyoto, Japan; 4 Junior Collage, Ryukoku University, Kyoto, Japan; 5 Ritsumeikan Medical Service Center, Kyoto, Japan; 6 Faculty of Psychology, Doshisha University, Kyoto, Japan; 7 Department of Life Design, Biwako Gakuin University, Higashiomi, Japan; 8 Department of Medical Life Systems, Doshisha University, Kyoto, Japan; 9 Department of Psychitary and Cognitive-Behavioral Medicine, Nagoya City University, Nagoya, Japan; 10 Population Health and Policy Research Unit, Medical Education Center, Kyoto University Graduate School of Medicine, Kyoto, Japan; 11 Department of Healthcare Epidemiology, Kyoto University Graduate School of Medicine / School of Public Health, Kyoto, Japan; 12 Department of Psychology, Vanderbilt University, Nashville, TN, USA; 13 Department of Scoial and Behavioral Sciences, School of Global Public Health, New York University, New York, NY, USA; 14 Department of Psychology, University of Exeter, Exeter, UK; 15 Population Health Scineces Institute, Newcastle University, Newcastle upon Tyne, UK; 16 Institute of Statistical Mathematics, Tachikawa, Tokyo, Japan; 17 National Center of Neurology and psychiatry/National Center for Cognitive Behavior Therapy and Research, Kodaira, Tokyo, Japan

**Keywords:** Depression & mood disorders, Child & adolescent psychiatry

## Abstract

**Background:**

Internet-based cognitive-behavioural therapy (iCBT) is effective for subthreshold depression. However, which skills provided in iCBT packages are more effective than others is unclear. Such knowledge can inform construction of more effective and efficient iCBT programmes.

**Objective:**

To examine the efficacy of five components of iCBT for subthreshold depression.

**Methods:**

We conducted an factorial trial using a smartphone app, randomly allocating presence or absence of five iCBT skills including self-monitoring, behavioural activation (BA), cognitive restructuring (CR), assertiveness training (AT) and problem-solving. Participants were university students with subthreshold depression. The primary outcome was the change on the Patient Health Questionnaire-9 (PHQ-9) from baseline to week 8. Secondary outcomes included changes in CBT skills.

**Findings:**

We randomised a total of 1093 participants. In all groups, participants had a significant PHQ-9 reduction from baseline to week 8. Depression reduction was not significantly different between presence or absence of any component, with corresponding standardised mean differences (negative values indicate specific efficacy in favour of the component) ranging between −0.04 (95% CI −0.16 to 0.08) for BA and 0.06 (95% CI −0.06 to 0.18) for AT. Specific CBT skill improvements were noted for CR and AT but not for the others.

**Conclusions:**

There was significant reduction in depression for all participants regardless of the presence and absence of the examined iCBT components.

**Clinical implication:**

We cannot yet make evidence-based recommendations for specific iCBT components. We suggest that future iCBT optimisation research should scrutinise the amount and structure of components to examine.

**Trial registration number:**

UMINCTR-000031307.

**Key messages**

**What is already known on this topic**

- Multicomponent internet-based cognitive-behavioural therapy (iCBT) is a promising intervention for subthreshold depression. However, optimal iCBT component and combination for subthreshold depression is yet unclear.

**What this study adds**

- This study was a large factorial trial to investigate optimal iCBT components or combinations for subthreshold depression. Results showed that college students with subthreshold depression reduced depressive symptoms on week 8, regardless of the presence or the absence of specific iCBT components of self-monitoring, behavioural activation, cognitive restructuring, assertiveness training, problem-solving.

**How this study might affect research, practice or policy**

- For investigating optimal iCBT component or combination for subthreshold depression, future research should assign adequate number of component for leaning each skills, and types of control conditions.

## Background

Subthreshold depression is highly prevalent in young populations.^1^ It not only negatively affects their quality of life but also puts them at risk for developing a major depressive disorder (MDD).^2 3^ Evidence-based psychotherapies, such as cognitive-behavioural therapy (CBT), have shown promise in reducing depressive symptom and preventing MDD in subthreshold depression.^4^ However, it has proven difficult to approach these populations as they may not seek professional help so willingly as more severely depressed individuals. In recent years, studies on the effects of internet-based CBT (iCBT) have accumulated and are promising,^5 6^ especially for these digitally native populations.

CBT is usually composed of a package of multiple skill components such as psychoeducation (PE), self-monitoring (SM), behavioural activation (BA), cognitive restructuring (CR), assertiveness training (AT) and problem-solving (PS) among others. In order to provide CBT efficiently to those who may be reluctant to seek active treatment, it is desirable to provide only the essentially effective components to make the help more accessible, efficient and effective. However, it is not clear which components or combinations of components are optimal for iCBT.

There are three methodological approaches to estimate specific efficacies of individual CBT skills. One is the traditional dismantling study, in which one component is added to or deleted from a package and the dismantled package is compared with the original one. Unfortunately, there have been only a handful of relatively small such studies and their conclusions remain inconclusive.^7 8^ A more recently emerging approach is the component network meta-analyses (cNMA), which decomposes and compares components in a network of randomised controlled trials (RCTs) representing various combinations of components.^9 10^ However, in cNMA, it is difficult to ensure homogeneity of components across the included RCTs and the number of effect modifiers that can be examined may be limited because different RCTs tend to measure different sets of baseline characteristics.^10^ A third approach that can overcome these limitations of power and inherent heterogeneity is a large-scale factorial trial, in which participants are randomly assigned to presence or absence of each component.^11^ This design can efficiently estimate the individual efficacy of components and their interactions, and thus screen for the most efficacious factors and combinations of factors while maintaining the integrity of a single large trial.^12^

In this study, we used the factorial design to explore the efficacy of various components of iCBT and their combinations among university students with subthreshold depression.

## Methods

### Trial design

The Healthy Campus Trial is a parallel-group, multicentre, open-label, stratified block randomised, factorial trial of five iCBT components including SM, CR, BA, AT and PS to examine the acute phase effects for subthreshold depression at 8 weeks and the long-term effects for depression prevention at 52 weeks. The design is a smartphone app-delivered component selection experiment with five experimental factors evaluated, each at two levels (presence vs absence), using a 32-condition orthogonal and balanced factorial design. This study is a report of the primary and secondary analyses of the acute phase effects. We have published the protocol for the whole study^13^ and hereby summarise the methods for the acute phase intervention up to 8 weeks. We followed the CONSORT guideline^14^ in preparing this report.

### Participants

We recruited undergraduate and graduate students, of any gender, aged between 18 and 39, presenting with subthreshold depression in four universities in Japan between September 2018 and May 2021. We first screened participants with the Patient Health Questionnaire-9 (PHQ-9),^15 16^ and invited randomly selected 10% of students who scored 4 or less, all those who scored between 5 and 9, and those who scored between 10 and 14 but scored 0 or 1 on its ninth item (suicidal ideation). Those who provided informed consent were administered the PHQ-9 again on randomisation: for the current analysis, we included those participants who scored 5 or higher on the PHQ-9 on randomisation. We promoted recruitment of participants by posting posters and brochures in each university and posting information on part-time job websites for university students. The participants had to own their own smartphone and to provide written informed consent. Additionally, they had to complete the psychoeducation (PE) component within 2 weeks after providing consent. We excluded students who could not understand the Japanese language and were currently receiving treatments for mental health problems. We also excluded candidates who scored 15 or more in total scores, or between 10 and 14 in total scores plus 2 or 3 on the ninth item (suicidal ideation), according to the PHQ-9 on screening.

### Interventions

Our smartphone app named ‘Resilience Training’ included six iCBT components of PE, SM, BA, CR, AT and PS. While PE was a constant component provided to all participants, and SM, BA, CR, AT and PS were experimental components for comparison between their presence and absence. All participants first received the PE lesson that provided psychoeducation about stress and the CBT model and emphasised the importance of self-checks through weekly PHQ-9 assessments.

After PE, the app system randomly assigned each participant to 1 of the 2ˆ5=32 combinations corresponding with presence or absence of SM, BA, CR, AT or PS. Online supplemental table 1 shows all the combinations of components. Each component consisted of a psychoeducational lesson explaining the rationale and steps for each cognitive or behavioural skill and homework sheets to practice the learnt skill. Each lesson was supposed to take 1 week to complete.

10.1136/ebmental-2022-300455.supp1 Supplementary data

SM provided the cognitive-behavioural model of reactions to the situations in terms of feelings, thoughts, behaviours and bodily responses. Participants learnt methods to monitor their reactions to situations and to understand how feelings, thoughts and behaviours interacted with each other through filling in mind maps. They were asked to complete at least one mind map from their daily life before proceeding to the next component.

CR provided psychoeducation of the relationship between thought and emotion, and worksheets for alternative thoughts. To help the participants broaden their views, CR offered three tools that help them to arrive at alternative thoughts through interactions with the characters.

BA explained the ‘outside-in’ principle of behaviour and provided worksheets for personal experiments to test out pleasurable activities, which were gamified in the form of an ‘action marathon.’

AT teaches participants the principle and methods of assertive communication. The participants learnt how to express their feelings assertively in specific situations.

PS presented systematic methods to clarify the problem and brainstorm solutions, with worksheets to write down the five-steps of structured problem solving. All participants received templated yet personalised encouragement e-mails to proceed with the programme (but without specific guidance for iCBT contents) from the study personnel.

When a participant scored 10 or higher on the PHQ-9 and its ninth item was 2 or higher for two consecutive weeks, we sent an email to advise them to contact the university mental services.

### Outcomes

The primary outcome was the change of depressive symptoms measured with the PHQ-9 at week 8 after randomisation. The PHQ-9 consists of 9 items from the major depressive episode diagnostic criteria in the Diagnostic and Statistical Manual of Mental Disorder, Fourth Edition,^17^ each rated between 0=not al all and 3=nearly every day.

The secondary outcomes were anxiety symptoms measured by the Generalised Anxiety Disorder-7 (GAD-7),^18^ CBT skills for each intervention component of this trial by the CBT Skills Scale,^19^ and the Presenteeism scale from the WHO Health and Work Performance Questionnaire (HPQ)^20^ at week 8 after randomisation.

### Sample size

We performed power calculations using the FactorialPowerPlan SAS Macro (available for free download at https://publichealth.nyu.edu/ioi). To detect an effect size (standardised mean difference (SMD)) of 0.20 for each component and interaction, at two-sided α=0.05 and β=0.10, a sample size of 1051 was necessary. To assure equal allocation to 32 combinations, recruiting 1088 participants (34 persons by 32 combinations) was deemed necessary. Although no increase for follow-up attrition was made, this was expected to be balanced by the increase in power from adjustment for baseline and use of repeated measures.

### Randomisation

We used permuted block randomisation, stratified by the university and the baseline PHQ-9 score (4 or less vs 5 or more). The random allocation sequence was generated by SAS PROC PLAN by an independent statistician to ensure a 1:1allocation to the presence or absence of each intervention factor. Participants were randomly assigned to zero to five experimental components, each of which received a different number of interventions (online supplemental table 1). Only the statistician and the principal investigator, who were not directly involved in participant recruitment, knew the block size. The smartphone application system automatically randomly assigned participants who had completed the PE lesson to each group. Thus, the allocation was concealed from the study personnel who enrolled the participants.

### Blinding

The participants and study personnel were not blinded to the intervention. Primary and secondary outcome measures were self-reports by the participant. The statistician analysing the dataset was kept blinded until the results were out.

### Statistical analyses

We used SAS V.9.4 (SAS Institute) for all analyses. Participants were analysed according to their randomisation group, including all participants randomised regardless of intervention actually received or study withdrawal, that is, on an intention-to-treat basis. We used the mixed-effects repeated-measures analyses (MMRM) to estimate the differences in mean change scores for presence vs absence of each component. The model included the subject as a random effect, and treatments (main effects and second-order interaction effects of the five components), week, treatment by week interaction, university, age and baseline PHQ-9 score as fixed effects. We calculated pre–post effect sizes by dividing the estimated mean changes from baseline to week 8 by the observed SD of baseline scores, and between-group effect sizes (SMD) at week 8 by dividing the estimated mean differences in change scores between groups by the observed SD of week 8 scores.

We used the same model for the GAD-7 and the CBT Skills Scale at week 8. Both scales were measured three times; baseline, week 4 and week 8. For the Presenteeism scale, we estimated the mean difference in change scores at week 8 using a general linear model, because it was measured only at two time points, baseline and week 8.

### Changes from the protocol

To recruit a broader range of subjects with subthreshold depression, we changed our original exclusion criteria of ‘10 or more points on the PHQ-9’ to 15 or more points, or 10 or more plus 2 or more points on item 9’ before enrolment of the first participant.

## Findings

### Baseline characteristics

**Figure 1:**
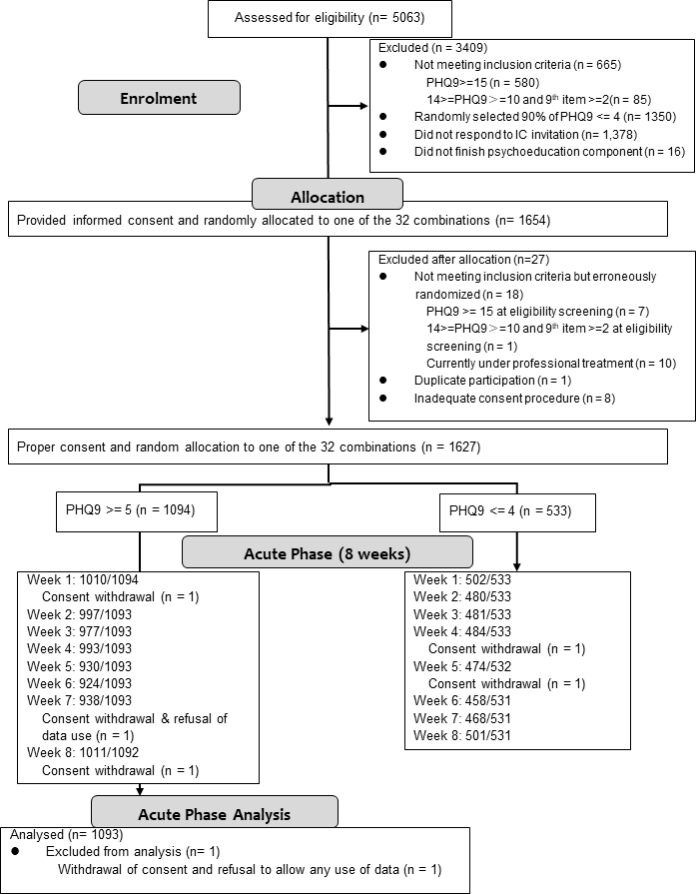
CONSORT diagram. IC, informed consent; PHQ-9: Patient Health Questionnaire-9.

Figure 1 shows the CONSORT flow diagram. Five thousand and sixty-three college students filled in the screening questionnaires. One thousand six hundred and twenty-seven participants were eligible and randomly assigned to each of the 32 possible combinations of components, of whom 1094 had a baseline PHQ-9 score of 5 or more and were subjects for the current study. One participant withdrew consent and refused to allow the data to be used, so we finally included 1093 in the current analyses.

Table 1 shows the participants' baseline characteristics. Their mean age was 21.7 (SD=3.03, range=18–39) and 58% were women. There were no major differences in baseline characteristics between the present and absent groups for each of the five intervention components.

**Table 1:** Baseline characteristics of total participants for acute phase analysis (N=1093) and by each component

	Total		Components																			
			SM				BA				CR				AT				PS			
			Presence		Absence		Presence		Absence		Presence		Absence		Presence		Absence		Presence		Absence	
N	1093		544		549		552		541		544		549		547		546		546		547	
Demographic	n	%	n	%	n	%	n	%	n	%	n	%	n	%	n	%	n	%	n	%	n	%
Age (M, SD)	21.6	3.03	21.7	3.05	21.6	3.01	21.7	2.98	21.5	3.08	21.7	3.11	21.6	2.94	21.6	3.23	21.6	2.82	21.6	3.27	21.6	2.78
Sex (female)	641	58%	319	59%	322	59%	326	59%	315	58%	315	58%	326	59%	323	59%	318	58%	330	60%	311	57%
Undergraduate	829	76%	406	75%	423	77%	415	75%	414	77%	422	78%	407	74%	414	76%	415	76%	415	76%	414	77%
Married	21	2%	10	2%	11	2%	8	1%	13	2%	9	2%	12	2%	9	2%	12	2%	9	2%	12	2%
Living alone	686	62%	353	65%	333	61%	356	64%	330	61%	346	64%	340	62%	344	63%	342	63%	341	62%	345	64%
Part-time employment	845	77%	413	76%	432	79%	437	79%	408	75%	421	77%	424	77%	426	78%	419	77%	426	78%	419	77%
Smoking regularly	68	6%	37	7%	31	6%	35	6%	33	6%	35	6%	33	6%	30	5%	38	7%	38	7%	30	6%
Drinking alcohol regularly	466	42%	229	42%	237	43%	237	43%	229	42%	233	43%	233	42%	239	44%	227	42%	224	41%	242	45%
Exercise regularly	666	61%	332	61%	334	61%	335	61%	331	61%	334	61%	332	60%	325	59%	341	62%	340	62%	326	60%
History of psychiatric/psychological treatment	130	12%	73	13%	57	10%	62	11%	68	13%	57	10%	73	13%	62	11%	68	12%	60	11%	70	13%
History of major depressive episode in past year (CIDI)	108	10%	60	11%	48	9%	55	10%	53	10%	58	11%	50	9%	49	9%	59	11%	56	10%	52	10%
Cognitive and behavioural skills	M	SD	M	SD	M	SD	M	SD	M	SD	M	SD	M	SD	M	SD	M	SD	M	SD	M	SD
SM skills	7.60	3.11	7.49	3.17	7.71	3.04	7.59	3.09	7.61	3.13	7.57	3.18	7.63	3.03	7.54	3.14	7.66	3.08	7.60	3.10	7.61	3.12
BA skills	8.42	3.43	8.42	3.46	8.42	3.40	8.56	3.42	8.28	3.43	8.59	3.55	8.25	3.30	8.57	3.42	8.27	3.43	8.31	3.67	8.52	3.16
CR skills	9.78	4.21	9.86	4.40	9.70	4.01	9.72	4.36	9.84	4.05	9.93	4.12	9.63	4.29	9.89	4.26	9.66	4.16	9.97	4.13	9.59	4.28
AT skills	9.31	3.49	9.13	3.54	9.48	3.45	9.27	3.40	9.35	3.59	9.33	3.63	9.28	3.36	9.36	3.54	9.25	3.45	9.05	3.48	9.56	3.49
PS skills	10.4	3.13	10.2	3.11	10.7	3.14	10.4	3.08	10.6	3.18	10.4	3.14	10.5	3.13	10.6	3.16	10.3	3.10	10.4	3.25	10.5	3.01
Clinical characteristics	M	SD	M	SD	M	SD	M	SD	M	SD	M	SD	M	SD	M	SD	M	SD	M	SD	M	SD
PHQ-9	8.10	2.76	8.21	2.88	7.99	2.64	8.15	2.82	8.04	2.70	8.06	2.69	8.14	2.84	8.17	2.77	8.03	2.76	8.04	2.70	8.15	2.83
GAD-7	6.40	3.33	6.28	3.15	6.52	3.51	6.64	3.32	6.16	3.33	6.45	3.26	6.34	3.40	6.51	3.37	6.29	3.29	6.42	3.21	6.37	3.45
Function	M	SD	M	SD	M	SD	M	SD	M	SD	M	SD	M	SD	M	SD	M	SD	M	SD	M	SD
WHO-HPQ Presenteeism	4.41	2.14	4.37	2.09	4.45	2.18	4.37	2.10	4.45	2.17	4.41	2.15	4.42	2.12	4.44	2.14	4.38	2.13	4.49	2.13	4.33	2.14

AT, assertiveness training; BA, behavioural activation; CIDI, Composite International Diagnostic Interview; CR, cognitive restructuring; GAD-7, Generalised Anxiety Disorder-7; PHQ-9, Patient Health Questionnaire-9; PS, problem-solving; SM, self-monitoring; WHO-HPQ, WHO-Health and Work Performance Questionnaire.

The summary statistics are shown either as the total number and proportion (n, %) or as the mean and its standard deviation (M, SD).

### Smartphone CBT use

Online supplemental table 2 shows the use of smartphone CBT. Participants spent an average of 27 min to complete each of the assigned components. Completion rates for each component ranged from 82% for PS to 91% for SM. Online supplemental table 3 shows the completion rates of the components when they were presented as the first through the fifth intervention after PE. While 93% of participants completed their first allocated component, the completion rate decreased with each step, with the fifth intervention component completed by 61% of those who were allocated to all five components.

### Primary analyses

**Figure 2:**
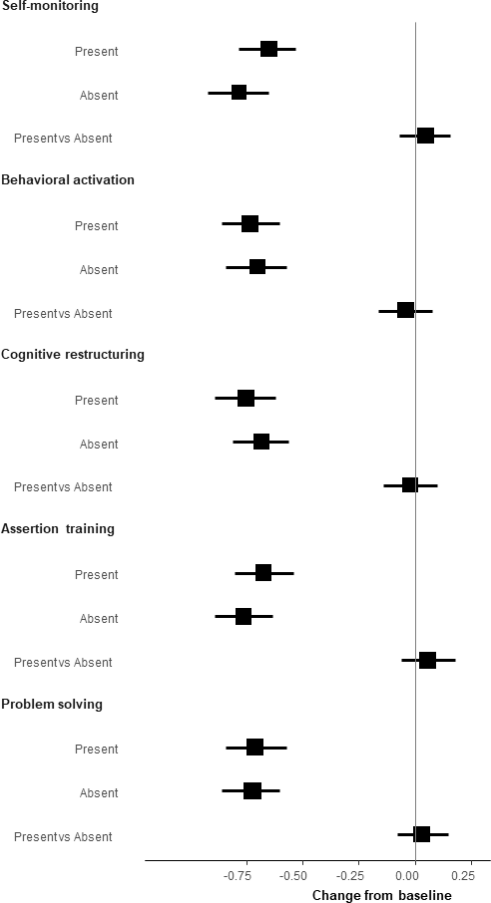
Effect size of the PHQ-9 change scores and their differences for each component at week 8. The effect size of change scores was calculated using the baseline SD, and the effect size for the differences between presence and absence was calculated using the week 8 SD. PHQ-9, Patient Health Questionnaire-9.

The primary outcome was available for 92% of the participants. Missing outcomes were accounted for through the MMRM under a missing-at-random assumptions. Table 2 shows estimated changes in the PHQ-9 scores from week 1 to week 8 and their model-based estimated differences in change scores for the presence or absence of each component at week 8. Figure 2 depicts standardised mean changes from baseline and their differences for the presence or abasence of each component. Online supplemental table 3 provides the unadjusted means and SDs of PHQ-9 scores at all time points.

**Table 2:** Analysis of main effects using the mixed-model L repeated measures analysis

Component	N*	Estimated least squares mean change scores of the PHQ-9†								Estimated differences of change scores (95% CI)	Effect size for Baseline-Week eight change scores‡ (95% CI)	Effect size for differences of change scores and their p values‡ (95% CI)	P value
		Week 1	Week 2	Week 3	Week 4	Week 5	Week 6	Week 7	Week 8				
SM													
Presence	544	−0.79	−1.31	−1.50	−1.86	−1.70	−1.75	−1.79	−1.88	0.19 (−0.29 to 0.67)	−0.65 (−0.78 to -0.53)	0.05 (−0.07 to 0.16)	0.44
Absence	549	−1.08	−1.34	−1.41	−1.48	−1.51	−1.67	−1.86	−2.07		−0.78 (−0.92 to -0.65)		
BA													
Presence	552	−0.85	−1.33	−1.42	−1.64	−1.60	−1.79	−1.85	−2.06	−0.17 (−0.65 to 0.31)	−0.73 (−0.86 to -0.60)	−0.04 (−0.16 to 0.08)	0.50
Absence	541	−1.01	−1.31	−1.49	−1.70	−1.61	−1.63	−1.79	−1.90		−0.70 (−0.84 to -0.57)		
CR													
Presence	544	−0.94	−1.05	−1.40	−1.60	−1.40	−1.47	−1.83	−2.02	−0.08 (−0.56 to 0.40)	−0.75 (−0.89 to -0.62)	−0.02 (−0.14 to 0.10)	0.74
Absence	549	−0.93	−1.60	−1.52	−1.74	−1.82	−1.95	−1.82	−1.94		−0.68 (−0.81 to -0.56)		
AT													
Presence	547	−0.75	−1.31	−1.30	−1.56	−1.71	−1.61	−1.73	−1.85	0.25 (−0.23 to 0.73)	−0.67 (−0.80 to -0.54)	0.06 (−0.06 to 0.18)	0.31
Absence	546	−1.12	−1.34	−1.62	−1.78	−1.50	−1.81	−1.91	−2.10		−0.76 (−0.89 to -0.63)		
PS													
Presence	546	−0.80	−1.38	−1.48	−1.63	−1.63	−1.74	−1.86	−1.91	0.14 (−0.34 to 0.62)	−0.71 (−0.84 to -0.57)	0.03 (−0.08 to 0.15)	0.56
Absence	547	−1.07	−1.27	−1.44	−1.71	−1.58	−1.69	−1.79	−2.05		−0.72 (−0.86 to −0.60)		

*In a factorial trial design, all 1093 participants included in the analysis were randomly assigned 1:1 to either presence or absence of each component.

†Minus value indicates depression reduction.

‡We calculated effect sizes (standardised mean differences) using the baseline SD for within-group change score and the week 8 SD for group differences.

AT, assertion training; BA, behavioural activation; CIDI, Composite International Diagnostic Interview; CR, cognitive restructuring; PHQ-9, Patient Health Questionnaire-9; PS, problem-solving; SM, self-monitoring

Depression was significantly reduced for all participants through 8 weeks, with pre–post effect sizes ranging between −0.65 and −0.78 at week 8. However, the presence of any of the components did not significantly enhance depression reduction, with between-group effect size point estimates ranging between −0.04 and 0.03. Second-order interactions between the components also showed no noteworthy effects (online supplemental table 5).

### Secondary analyses

Online supplemental table 6 shows estimated changes in the GAD-7 score, five CBT skills and presenteeism. In all groups, the participants’ anxiety was reduced from baseline to week 8: however, there was no significant difference in anxiety reduction between participants undertaking or not undertaking each component. There were similar tendencies for presenteeism. In CBT Skills, CR and AT skills were significantly increased among participants receiving the corresponding component in comparison with those not receiving it. None of the participants presented with serious adverse events.

## Discussion

University students with subthreshold depression showed substantial overall reduction in depressive symptoms during the 8-week intervention period of the factorial trial using the smartphone iCBT consisting of five iCBT components of SM, CR, BA, AT and PS. However, we could not find a difference in depression reduction between the presence and absence of the examined components. We found the same for the secondary outcomes of anxiety and presenteeism. We were, therefore, unable to determine the optimal components or combinations of components of iCBT based on the present results.

Adherence rates for each component ranged from 82% to 91%, and most participants engaged well with the assigned interventions, except when they were provided as the fourth or the fifth experimental component of lengthier intervention groups. In terms of CBT skills changes, specific skill improvements for the component were found for CR and AT. In contrast, this was not evident for SM, BA and PS. The CR and AT components in our smartphone app taught very specific skills in CR and AT that were probably new to the participants, whereas SM and BA skills may have appeared less particular and something one would practice when one’s depression becomes less severe. The reason why PS skills showed decrease, regardless of the presence or absence of the PS session, is less clear.

Several factorial trials of psychological and behavioural complex interventions have been conducted and their results are just starting to be reported.^21^ Surprisingly, many studies have failed to find expected effects of specific components or their interactions for various interventions including smoking cessation^22^ and iCBT programmes for depression,^23^ cancer recurrence fear^24^ or alcohol consumption,^25^ even when their sample sizes were fairly large.

By contrast, in an individual patient data cNMA (IPD-cNMA) study of iCBT for depression, BA was found to be an effective component, while CR was not: PS and AT appeared promising in point estimates but their credible intervals ranged widely from beneficial to harmful.^10^

The current factorial trial failed to find any specific efficacies of SM, BA, CR, AT or PS. We can only speculate the possible reasons for our failure to detect specific efficacies. First the participants may not have been most suited to detect meaningful effects of specific interventions of iCBT. A recent IPD-MA of internet-based interventions for subthreshold depression showed that the effect was greater for people with higher baseline severity and whose age was older.^6^ Another IPD-NMA found the iCBT had greater effects when the intervention was guided and the participants had greater baseline severity.^26^ Our iCBT intervention was guided, but the participants had only subthreshold depressive symptoms and were young. While our smartphone app was built for university students with subthreshold depression, these participants’ characteristics may have made it difficult for the trial to detect signals. In addition, many of the participants in the current study were recruited through the university’s part-time job advertisement sites where, in addition to ordinary part-time jobs for students, participants in various experiments conducted by the faculty members are recruited. These participants may have had less severe depressive symptoms and/or may have been less motivated to learn the CBT skills even when they reported subthreshold depressive symptoms.

Second, an observed reduction in depression over time in this study may be attributable to the natural course of depression; the constant component effect; and specific effects of the experimental components. It is possible that the constant component in our factorial trial was so strong and the additional incremental effect of the experimental components was very small. The constant component was a substantial intervention: All the participants received the PE, personalised encouragement emails to proceed with the programme and filled in the PHQ-9 every week, even when they were not assigned to any of the specific CBT skills. In fact, the pre–post effect sizes observed for presence or absence of each component in our smartphone app (−0.65 to −0.78, table 2) compares favourably with the active arms in the four positive RCTs of iCBT for subthreshold depression in the general population (pooled pre–post effect size: −0.93, 95% CI −1.53 to −0.33) and is superior to their control conditions (−0.33, 95% CI –0.59 to −0.07).^27–30^

By contrast, the experimental intervention in this study may have had too many components, each of which required a certain amount of time and effort of the participants. Thus, the additional burden required of those allocated to four or five components may have been too large compared with those assigned to one or two components, and this difference may have blurred the potential differences among the components, even though in our trial we had tried to balance the ordering of the components. This contrast is easy to see when we imagine a factorial trial of three elements whose presence or absence does not necessarily require additional efforts (eg, when an advertisement is written with many vs few words in large vs small fonts in red vs black colours) and another factorial trial where each element would require a substantial commitment of the participants. In other words, the same components when included as the fourth or fifth experimental element may have been unable to exert its full effects. A factorial trial with three or fewer components could have been more sensitive to signals. The changes indeed were smaller in later weeks of the intervention than in the earlier 2 weeks. A corollary of having many components was that their random assignment resulted in a loss of continuity in the intervention. Even when multiple components are used in clinical practice, clinicians pay attention to the buildup and relationships among the components, such as introducing CR methods based on the results of BA and emphasising continuation of behavioural experiments while working on a different skill in later sessions. The programme used in this study did not have such inter-component relationships: in other words, there was no explanation of accumulation of knowledge and skills when the participants went through three or four or five CBT lessons and they may have felt lost in the programme especially with regards to the skills taught later in the programme, which also may have contributed to the non-differentiation among the components.

An external circumstance in this trial was not very favourable for learning iCBT either. We recruited more than half of our participants during the COVID-19 pandemic when various containment measures including school closures, stay-at-home orders and travel restrictions were in effect. With reduced social activities, participants may not have had the opportunity to practice and use their CBT, especially behavioural, skills. For example, some participants commented that they had no occasion to practice their assertion skills that they had learnt in the programme.

Nevertheless, the study as planned and as executed had several strengths. First, it was strictly designed as a factorial trial with a prepublished protocol.^13^ Second it was rigorously conducted with adequate allocation concealment, little deviation from the intended interventions and very small lost to follow-up, and analysed according to the predefined protocol. Lastly, the study had a very large sample size.

## Conclusions and implications

This is the first factorial trial that has sought to optimise five major components of CBT for subthreshold depression. Contrary to our expectations, we did not find any particular component or combination of components superior. Clinically, therefore, at this time we cannot make an evidence-based decision to recommend a specific component for iCBT for subthreshold depression. By contrast, the study has many research implications. Future iCBT optimisation trials should assign only the number of components that participants can adequately engage with, provide continuity among multiple components, and consider multiple types of control conditions to estimate the effects of interventions. The search for more effective iCBT components and their optimal combinations should be continued to maximise their benefits with minimum burden on their users.

## Data Availability

Data are available on reasonable request. After the publication of the primary findings, the deidentified and completely anonymised individual participant-level dataset will be posted on the UMIN-ICDR website (http://www.umin.ac.jp/icdr/index-j.html) for access by qualified researchers.
