# Supplementary material for: Components of smartphone cognitive-behavioural therapy for subthreshold depression among 1093 university students: a factorial trial
Source: Evid Based Ment Health. 2022 May 16;25(e1):e18–25. doi: 10.1136/ebmental-2022-300455 (PMC9811098; doi:10.1136/ebmental-2022-300455)
Supplement: Supplementary data [file ebmental-2022-300455supp001.pdf]

Supplementary file to "Components of smartphone cognitive-behavioural therapy for subthreshold depression: A fully factorial randomized controlled trial"

eTable 1. Combinations of iCBT component

	PE	SM	CR	BA	AT	PS	Order								n
C1	1	1	1	1	1	1	**PE**	**AT**	**PS**	**SM**	**CR**	**BA**			17
C2	1	1	1	1	1	1	**PE**			**SM**	**BA**	**CR**	**AT**	**PS**	17
C3	1	1	1	1	1	0	**PE**			**SM**	**CR**	**BA**	**AT**		17
C4	1	1	1	1	1	0	**PE**	**AT**		**SM**	**BA**	**CR**			17
C5	1	1	1	1	0	1	**PE**		**PS**	**SM**	**CR**	**BA**			17
C6	1	1	1	1	0	1	**PE**			**SM**	**BA**	**CR**		**PS**	17
C7	1	1	1	1	0	0	**PE**			**SM**	**CR**	**BA**			17
C8	1	1	1	1	0	0	**PE**			**SM**	**BA**	**CR**			17
C9	1	1	1	0	1	1	**PE**			**SM**	**CR**		**AT**	**PS**	17
C10	1	1	1	0	1	1	**PE**	**AT**	**PS**	**SM**	**CR**				17
C11	1	1	1	0	1	0	**PE**			**SM**	**CR**		**AT**		17
C12	1	1	1	0	1	0	**PE**	**AT**		**SM**	**CR**				17
C13	1	1	1	0	0	1	**PE**			**SM**	**CR**			**PS**	17
C14	1	1	1	0	0	1	**PE**		**PS**	**SM**	**CR**				17
C15	1	1	1	0	0	0	**PE**			**SM**	**CR**				17
C16	1	1	1	0	0	0	**PE**			**SM**	**CR**				17
C17	1	1	0	1	1	1	**PE**			**SM**		**BA**	**AT**	**PS**	17
C18	1	1	0	1	1	1	**PE**	**AT**	**PS**	**SM**		**BA**			17
C19	1	1	0	1	1	0	**PE**			**SM**		**BA**	**AT**		17
C20	1	1	0	1	1	0	**PE**	**AT**		**SM**		**BA**			17
C21	1	1	0	1	0	1	**PE**			**SM**		**BA**		**PS**	17
C22	1	1	0	1	0	1	**PE**		**PS**	**SM**		**BA**			17
C23	1	1	0	1	0	0	**PE**			**SM**		**BA**			17
C24	1	1	0	1	0	0	**PE**			**SM**		**BA**			17
C25	1	1	0	0	1	1	**PE**			**SM**			**AT**	**PS**	17
C26	1	1	0	0	1	1	**PE**	**AT**	**PS**	**SM**					17
C27	1	1	0	0	1	0	**PE**			**SM**			**AT**		17
C28	1	1	0	0	1	0	**PE**	**AT**		**SM**					17
C29	1	1	0	0	0	1	**PE**			**SM**				**PS**	17
C30	1	1	0	0	0	1	**PE**		**PS**	**SM**					17
C31	1	1	0	0	0	0	**PE**			**SM**					17
C32	1	1	0	0	0	0	**PE**			**SM**					17
C33	1	0	1	1	1	1	**PE**				**CR**	**BA**	**AT**	**PS**	17
C34	1	0	1	1	1	1	**PE**	**AT**	**PS**		**BA**	**CR**			17
C35	1	0	1	1	1	0	**PE**	**AT**			**CR**	**BA**			17

C36	1	0	1	1	1	0	**PE**				**BA**	**CR**	**AT**		17
C37	1	0	1	1	0	1	**PE**				**CR**	**BA**		**PS**	17
C38	1	0	1	1	0	1	**PE**		**PS**		**BA**	**CR**			17
C39	1	0	1	1	0	0	**PE**				**CR**	**BA**			17
C40	1	0	1	1	0	0	**PE**				**BA**	**CR**			17
C41	1	0	1	0	1	1	**PE**				**CR**		**AT**	**PS**	17
C42	1	0	1	0	1	1	**PE**	**AT**	**PS**		**CR**				17
C43	1	0	1	0	1	0	**PE**				**CR**		**AT**		17
C44	1	0	1	0	1	0	**PE**	**AT**			**CR**				17
C45	1	0	1	0	0	1	**PE**				**CR**			**PS**	17
C46	1	0	1	0	0	1	**PE**		**PS**		**CR**				17
C47	1	0	1	0	0	0	**PE**				**CR**				17
C48	1	0	1	0	0	0	**PE**				**CR**				17
C49	1	0	0	1	1	1	**PE**					**BA**	**AT**	**PS**	17
C50	1	0	0	1	1	1	**PE**	**AT**	**PS**			**BA**			17
C51	1	0	0	1	1	0	**PE**					**BA**	**AT**		17
C52	1	0	0	1	1	0	**PE**	**AT**				**BA**			17
C53	1	0	0	1	0	1	**PE**					**BA**		**PS**	17
C54	1	0	0	1	0	1	**PE**		**PS**			**BA**			17
C55	1	0	0	1	0	0	**PE**					**BA**			17
C56	1	0	0	1	0	0	**PE**					**BA**			17
C57	1	0	0	0	1	1	**PE**						**AT**	**PS**	17
C58	1	0	0	0	1	1	**PE**	**AT**	**PS**						17
C59	1	0	0	0	1	0	**PE**						**AT**		17
C60	1	0	0	0	1	0	**PE**	**AT**							17
C61	1	0	0	0	0	1	**PE**							**PS**	17
C62	1	0	0	0	0	1	**PE**		**PS**						17
C63	1	0	0	0	0	0	**PE**								17
C64	1	0	0	0	0	0	**PE**								17

Note: For the overall Healthy Campus Trial, 64 groups were constructed by combining five components and one type of reordering to see the order effect of BA and CR. In the present study, the analysis was conducted as 32 groups to examine the effects of the five components only.
AT: assertiveness training, BA: behavioral activation, CR: cognitive restructuring, PS: problem solving, SM: self-monitoring

eTable 2. iCBT use and retention by each component

			Components																			
			SM				BA				CR				AT				PS			
	Total		Presence		Absence		Presence		Absence		Presence		Absence		Presence		Absence		Presence		Absence	
N	1093		544		549		552		541		544		549		547		546		546		547	
	M	SD	M	SD			M	SD			M	SD			M	SD			M	SD		
Total access time per component for all randomised (minutes)	24.8	34.8	17.8	15.1			18.2	27.5			29.1	88.7			30.2	19.9			26.1	21.4		
Total access time per component for completers (minutes)	27.3	36.2	18.8	14.6			20.9	28.7			33.0	95.7			33.6	18.5			30.5	19.2		
Worksheets completed			3.83	6.29			14.9	125			3.38	2.43			1.79	1.24			2.17	1.84		
	n	%	n	%	n	%	n	%	n	%	N	%	n	%	n	%	n	%	n	%	n	%
Completed component			497	91%			466	84%			459	84%			455	83%			446	82%		
Completed assessment																						
Week 1	1010	92%	506	93%	504	92%	520	94%	490	91%	506	93%	504	92%	503	92%	507	93%	506	93%	504	93%
Week 2	997	91%	499	92%	498	91%	502	91%	495	91%	500	92%	497	91%	503	92%	494	90%	492	90%	505	93%
Week 3	977	89%	493	91%	484	88%	491	89%	486	90%	484	89%	493	90%	493	90%	484	89%	486	89%	491	91%
Week 4	993	91%	489	90%	504	92%	498	90%	495	91%	500	92%	493	90%	493	90%	500	92%	490	90%	503	93%
Week 5	930	85%	471	87%	459	84%	466	84%	464	86%	465	85%	465	85%	463	85%	467	86%	461	84%	469	87%
Week 6	924	85%	466	86%	458	83%	470	85%	454	84%	459	84%	465	85%	456	83%	468	86%	463	85%	461	85%
Week 7	938	86%	472	87%	466	85%	468	85%	470	87%	465	85%	473	86%	469	86%	469	86%	465	85%	473	87%
Week 8	1011	92%	502	92%	509	93%	507	92%	504	93%	501	92%	510	93%	505	92%	506	93%	503	92%	508	94%

AT: assertiveness training, BA: behavioral activation, CR: cognitive restructuring, PS: problem solving, SM: self-monitoring

eTable 3. iCBT use and retention by component order

	Component order									
	1st		2nd		3rd		4th		5th	
n	1060		884		545		208		36	
	n	%	n	%	n	%	n	%	N	%
Completed component	988	93%	747	85%	420	77%	146	70%	22	61%
	M	SD	M	SD	M	SD	M	SD	M	SD
Total access time per component for all randomised	27.2	20.2	23.8	23.5	23.9	64.2	20.9	23.7	11.9	16.7
Total access time per component for completers	27.7	19.9	26.0	23.1	28.8	71.8	28.4	23.7	19.5	17.7

eTable 4. Unadjusted means and change scores for PHQ-9 by each component (N = 1,093)

			Component																		
			SM				BA				CR				AT				PS			
	Total		Presence		Absence		Presence		Absence		Presence		Absence		Presence		Absence		Presence		Absence	
PHQ9 score	M	SD	M	SD	M	SD	M	SD	M	SD	M	SD	M	SD	M	SD	M	SD	M	SD	M	SD
Baseline	8.10	2.76	8.21	2.88	7.99	2.64	8.15	2.82	8.04	2.70	8.06	2.69	8.14	2.84	8.17	2.77	8.03	2.76	8.04	2.70	8.15	2.83
Week 1	7.20	3.51	7.40	4.10	7.00	3.63	7.32	3.66	7.08	4.07	7.17	3.77	7.23	3.98	7.41	3.89	6.99	3.85	7.29	3.94	7.12	3.80
Week 2	6.81	3.53	6.85	3.88	6.77	3.90	6.85	3.84	6.77	3.94	7.08	4.12	6.54	3.62	6.84	3.85	6.78	3.92	6.70	3.92	6.92	3.84
Week 3	6.67	3.78	6.68	4.14	6.66	4.10	6.73	4.15	6.61	4.10	6.69	4.17	6.66	4.08	6.90	4.13	6.43	4.10	6.61	4.20	6.73	4.05
Week 4	6.47	3.82	6.30	4.04	6.63	4.14	6.51	4.16	6.42	4.03	6.55	4.16	6.38	4.03	6.62	4.20	6.31	3.99	6.44	4.19	6.49	4.00
Week 5	6.55	3.90	6.49	4.13	6.62	4.44	6.66	4.23	6.45	4.34	6.78	4.41	6.33	4.15	6.51	4.23	6.60	4.34	6.51	4.31	6.60	4.26
Week 6	6.40	4.01	6.41	4.36	6.39	4.34	6.34	4.31	6.46	4.39	6.60	4.34	6.21	4.35	6.56	4.45	6.25	4.25	6.36	4.35	6.44	4.35
Week 7	6.28	3.99	6.33	4.34	6.23	4.26	6.34	4.40	6.23	4.20	6.26	4.29	6.30	4.31	6.40	4.30	6.16	4.30	6.13	4.41	6.43	4.18
Week 8	6.16	4.11	6.30	4.39	6.01	4.15	6.12	4.34	6.19	4.20	6.12	4.27	6.19	4.28	6.29	4.32	6.02	4.22	6.20	4.45	6.11	4.09
Baseline-Week 8 change score	-1.93	4.13	-1.87	4.12	-2.00	3.88	-2.00	4.05	-1.87	3.96	-1.95	3.90	-1.92	4.11	-1.90	3.94	-1.97	4.07	-1.83	4.02	-2.04	3.99
Difference of change score			0.13				-0.13				-0.02				0.08				0.21			

AT: assertiveness training, BA: behavioral activation, CR: cognitive restructuring, PHQ-9: Patient Health Questionnaire-9, PS: problem solving, SM: self-monitoring

BMJ Publishing Group Limited (BMJ) disclaims all liability and responsibility arising from any reliance placed on this supplemental material which has been supplied by the author(s)

eTable 5. Interaction by each component

Interaction	Estimate	95% CI
SM*BA	0.04	-0.92 to 0.99
SM*CR	0.03	-0.92 to 0.99
SM*AT	0.09	-0.87 to 1.04
SM*PS	-0.99	-1.95 to -0.04
BA*CR	0.43	-0.53 to 1.38
BA*AT	0.25	-0.71 to 1.21
BA*PS	0.06	-0.94 to 0.97
CR*AT	0.39	-0.57 to 1.34
CR*PS	-0.34	-1.30 to 0.62
AT*PS	-0.06	-1.02 to 0.90

AT: assertiveness training, BA: behavioral activation, CR: cognitive restructuring, PS: problem solving, SM: self-monitoring

eTable 6. Analysis of secondary outcomes

				CBT Skills*											
		GAD7*		SM		BA		CR		AT		PS		Presenteeism**	
Component	n	Week 8	Difference (95% CI)	Week 8	Difference (95% CI)	Week 8	Difference (95% CI)	Week 8	Difference (95% CI)	Week 8	Difference (95% CI)	Week 8	Difference (95% CI)	Week 8	Difference (95% CI)
SM															
Presence	544	-0.88	0.39	0.77	0.06									0.30	0.03
Absence	549	-1.27	(-0.05 to 0.82)	0.70	(-0.28 to 0.40)									0.35	(-0.51 to 0.57)
BA															
Presence	552	-1.13	-0.10			1.13	0.04							0.34	0.34
Absence	541	-1.02	(-0.54 to 0.33)			1.10	(-0.36 to 0.43)							0.32	(-0.20 to 0.88)
CR															
Presence	544	-0.99	0.17					1.15	0.36					0.31	0.05
Absence	549	-1.16	(-0.26 to 0.61)					0.80	(0.03 to 0.67)					0.34	(-0.49 to 0.59)
AT															
Presence	547	-0.95	0.25							1.26	0.35			0.40	0.39
Absence	546	-1.20	(-0.18 to 0.68)							0.91	(0.02 to 0.68)			0.25	(-0.15 to 0.93)
PS															
Presence	546	-1.06	0.02									-0.75	0.10	0.44	0.32
Absence	547	-1.08	(-0.41 to 0.46)									-0.85	(-0.16 to 0.36)	0.21	(-0.21 to 0.87)

*Estimated least squares mean change scores

**Estimated change scores

AT: assertiveness training, BA: behavioral activation, CR: cognitive restructuring, GAD-7: Generalized Anxiety Disorder-7, PS: problem solving, SM: self-monitoring, Presenteeism: WHO Health and Work Performance Questionnaire-presenteeism scale

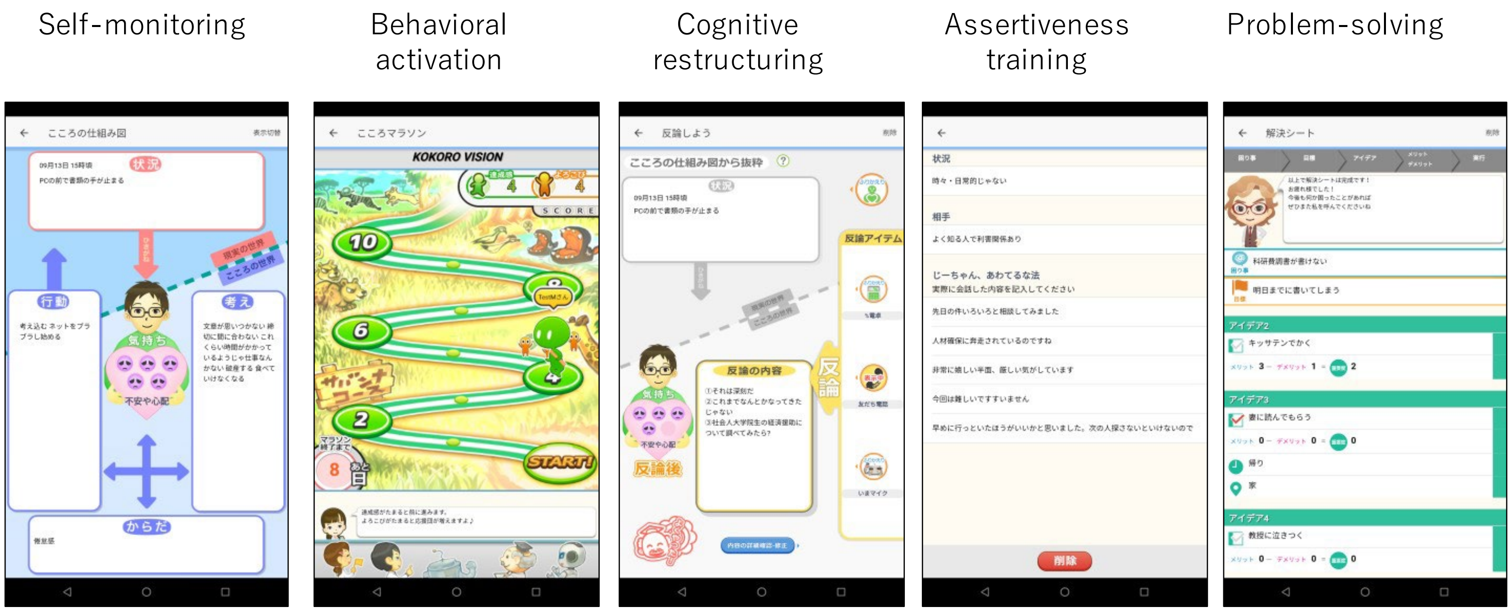

eFigure. Screenshots from each component of iCBT app “Resilience Training”
